# Two Cases of Trismus Nascentium, Which Recovered under the Use of Cannabis Indica

**Published:** 1853-12

**Authors:** P. C. Gaillard

**Affiliations:** Charleston, S. C.


*Two cases of Trismus Nascentium, which recovered under the use of Cannabis Indica.* Reported to the Medical Society of S. Carolina, Oct. 1st., 1853, by P. C. GAILLARD, M. D., Charleston, S. C.

I am induced to report the following case, with some details, in consequence of its unusual termination in recovery. " Trismus of new born infants," says Romberg, " is the most absolutely fatal of all children's diseases. Cases in which a cure has been effected, must be received with great suspicion, unless the diagnosis be verified by very accurate details." With us, the disease is, unfortunately, too common to allow us to be ignorant of its characteristic features ; and our experience teaches us that the German professor has not exaggerated its fearful mortality. But I am happy to be able to report another case of recovery, under the same treatment, which occurred in the practice of my friend, Dr. De Saussure, during the month of March, 1853. The details of this case, furnished by Dr. DeS., will be found below, (case 2.) Time and further experience must determine whether we possess in cannabis an agent capable of controlling the disease; or whether the cases which follow must be reckoned amongst those fortunate accidents, or coincidences, which too often mislead us in estimating the value of therapeutic agents.

*Case* 1.—Rachael, a negress, æt. 38, of good constitution, was delivered of her eleventh child on Saturday, 23d July, 1853. The cord fell on the sixth day, and the child, a fine robust boy, did well until Tuesday, August 2d, when, towards evening, it was observed to be fretful and to nurse with difficulty. During the night it lost the power of taking the breast, and paroxysms of spasm became evident. I saw it the next day, August 3d. The child was lying on the mother's lap.—Paroxysms of spasm could be readily brought on by blowing into the face, or by attempting to introduce the finger or a spoon into the mouth. During the paroxysms, the muscles of the face were contracted so as to produce the characteristic expression; the lips prominent; the jaws fixed and slightly separated; the tongue pressed against and filling the narrow space between them; the nose compressed; the forehead wrinkled longitudinally; and the child uttered the grunting, whining cry so peculiar in this disease. The muscles of the back and arms were rigid and the fingers were forcibly shut upon the palms of the hands.

The navel had healed; there were no signs of irritation about it. The examination of the head showed that the occipital bone was in its normal position; the parietals did not override, or in the slightest degree overlap its anterior border. The child was still able to swallow a little; for, although whenever the milk with which the mother fed it was poured into the mouth, the greater part was forced out by the spasmodic action induced by its introduction, yet after a time a little was swallowed.

The following directions were given: the child to be constantly fed, while awake, with milk drawn from the mother's breast and poured into the mouth; large warm poultices to be kept constantly to the abdomen; a warm bath to be given twice a day, and a teaspoonful of the following mixture to be administered every two hours: Tinct. Cannabis Indica ʒii, Camphor Water ℥ii.

On the 4th no change. On the 5th the spasms were reported as having increased in frequency and severity; the medicine was directed to be given every hour and a half. On the 6th and 7th no appreciable change; the treatment was assiduously prosecuted. On the 8th the dose of the medicine was increased: ʒii. of the tincture of cannabis to ℥ii. of camphor water, a teaspoonful every hour. On the 9th the mother said that the child had nursed a little twice when she forced the nipple into its mouth. The spasms of some of the muscles were, however, still very violent, as I could with difficulty, and only by using considerable force, extend the fingers, which were forcibly contracted on the palm. On the 10th the child took nourishment more freely by the spoon. On the 11th the spasms were rather less frequent, and not so severe; the rigidity was less, and the child nursed twice. The improvement continued progressively. On the 21st the spasms were very short, and the child took the breast readily when placed to its lips. On the 24th the mother reported that there had been no paroxysm of spasm for 24 hours, and that the child nursed freely. From the 16th, the intervals between the doses of medicine were gradually increased, and it was continued, three doses a day, for some days after the disease had disappeared. The child has continued well up to this time, October 1st.

*Case* 2.—Reported by H. W. DESAUSSURE, M. D. Priscilla, a stout, healthy negress, æt. 26, was delivered of her third child on the 13th March, 1853. The infant was a large healthy looking male. The cord was detached on the fifth day ; on the seventh day the child was noticed as unusually fretful, and passed several greenish, watery stools ; on the night of the eighth day, it was fretful, restless, scarcely slept at all, and was unable to move, although apparently anxious to do so. I saw it on the morning of the 9th day, the 22nd of March. It was lying in the nurse's arms, with the features contracted, the brows corrugated, the muscles of the neck and back rigid and hard, the arms and legs flexed, so as to be extended with difficulty; the hands contracted, with the thumbs drawn tightly upon the palms; the jaws not very tightly closed—the little finger could be introduced into the mouth by a little effort ; deglutition was possible, but the child could not nurse. The navel was not entirely healed, but the ulceration left by the dropping of the cord looked healthy. The child was ordered to be placed in a warm bath, a large poultice applied to the abdomen, and a teaspoonful of the following mixture given every two hours, viz : ℞ Tinct, ℥ss. ; Syr. wild cherry, ℥iss. ; and the mother's milk to be given by teaspoonfuls frequently. On the 23d there was aggravation of all the symptoms ; the jaws were so firmly closed that a teaspoon was with difficulty introduced into the mouth ; deglutition was extremely difficult ; the arms, hands and legs were more firmly flexed ; opisthotonos was present to a great degree ; the slightest motion, a breath of air upon the face, even touching the lips with the finger, was sufficient to bring on general spasms, accompanied by the peculiar whimpering cry so characteristic of this disease. The head was carefully examined ; no depression of the occipital bone could be detected. The same treatment was continued, and a teaspoonful of the mixture was given every hour. From the 24th to the 28th, the intensity of the disease gradually increased, until it seemed impossible that the child could survive each spasmodic paroxyism. It was with great difficulty, and only by patient perseverance, that either milk or medicine could be swallowed. All general treatment was now abandoned. A dose of the cannabis mixture was ordered to be given every half hour, and as much milk given as the infant could be made to swallow. On the 28th, 29th, 30th, and 31st March, half an ounce of tinct. cannabis was administered every twenty-four hours. On the 1st of April symptoms of amendment began to be visible ; the spasms were less frequent, and less easily excited ; the rigidity of the arms, hands and legs, was less ; and deglutition was effected with less difficulty. This improvement gradually but steadily progressed ; the quantity of cannabis was proportionately decreased, and by the 10th of April, the child was entirely convalescent. It is now a fine, healthy infant.—. *Charleston Med. and Surg. Journal.*

